# Glucose metrics and device satisfaction in adults with type 1 diabetes using different treatment modalities: a multicenter, real-world observational study

**DOI:** 10.1007/s00592-024-02381-3

**Published:** 2024-10-10

**Authors:** S. Di Molfetta, A. Rossi, R. Gesuita, A. Faragalli, A. Cutruzzolà, C. Irace, N. Minuto, D. Pitocco, F. Cardella, C. Arnaldi, A. Frongia, E. Mozzillo, B. Predieri, P. Fiorina, F. Giorgino, V. Cherubini

**Affiliations:** 1 Section of Internal Medicine, Endocrinology, Andrology and Metabolic Diseases, Department of Precision and Regenerative Medicine and Ionian Area, University of Bari Aldo Moro, Bari, 70124 Italy; 2 IRCCS Ospedale Galeazzi-Sant’Ambrogio, Milan, 20157 Italy; 3 Department of Biomedical and Clinical Sciences, University of Milan, Milan, 20122 Italy; 4 Center of Epidemiology, Biostatistics and Medical Information Technology, Polytechnic University of Marche, Ancona, 60020 Italy; 5 Department of Clinical and Experimental Medicine, University Magna Graecia, Catanzaro, 88100 Italy; 6 Department of Health Science, University Magna Graecia, Catanzaro, 88100 Italy; 7 Department of Pediatrics, IRCCS Istituto Giannina Gaslini, Genoa, 16147 Italy; 8 Diabetes Care Unit, UOSD Diabetologia, Fondazione Policlinico Universitario Agostino Gemelli, IRCCS, Rome, 00168 Italy; 9 Department of Pediatrics, University of Palermo, Palermo, 90133 Italy; 10 UOS Diabetologia Pediatrica ASL Viterbo, Viterbo, 01100 Italy; 11 Diabetes Unit, Ospedale Brotzu, Cagliari, 09134 Italy; 12 Department of Translational Medical Science, Section of Pediatrics, Regional Center of Pediatric Diabetes, Federico II University of Naples, Naples, 80138 Italy; 13 Department of Medical and Surgical Sciences of the Mother, Children and Adults - Pediatric Unit, University of Modena and Reggio Emilia, Modena, 41124 Italy; 14 Division of Endocrinology, ASST Fatebenefratelli-Sacco, Milan, 20121 Italy; 15 Department Biomedical and Clinical Sciences “L. Sacco”, University of Milan, Milan, 20122 Italy; 16 International Center for T1D - Pediatric Clinical Research Center Romeo ed Enrica Invernizzi, Milan, 20157 Italy; 17 Nephrology Division, Boston Children’s Hospital, Harvard Medical School, Boston, MA USA; 18 Department of Women’s and Children’s Health, Salesi Hospital, Ancona, 60123 Italy

**Keywords:** Type 1 diabetes, Adults, Treatment, Continuous subcutaneous insulin infusion, Hybrid closed loop, Time in range, Device satisfaction

## Abstract

**Aims:**

To evaluate glucose metrics, device satisfaction and diabetes impact in adults with type 1 diabetes using different treatment modalities in a real-life setting in Italy.

**Methods:**

This was a multicentre, nationwide, cross-sectional study. Candidates were consecutively evaluated for eligibility during their routine medical visit at the diabetes centre. Researchers collected comprehensive demographic, socioeconomic, anamnestic and clinical data, and administered the Diabetes Impact and Device Satisfaction scale.

**Results:**

From 2021 to 2022, a total of 428 subjects, 45% males, with a median age of 32 years (IQR 23–47) were recruited in 11 participating centres from all over Italy. No differences in age, physical activity, and diabetes impact were found for the different treatment modalities. HCL/AHCL and SAP groups reported higher device satisfaction vs. MDI + SMBG and MDI + CGM (*p* < 0.001). Subjects treated with HCL/AHCL exhibited significantly higher TIR and significantly lower time spent in hypoglycemia level 1, time spent in hyperglycemia, CV and GMI compared to MDI + CGM, and significantly higher TIR and significantly lower time spent in hypoglycemia level 2, time spent in hyperglycemia, and CV compared to SAP. Significant reduction in hypoglycemia level 2 was also found with PLGM compared to SAP. High education attainment was associated with optimal metabolic control.

**Conclusion:**

Real-life use of advanced technologies for type 1 diabetes is associated with improved glucose metrics and device satisfaction. Education level also contributes to success of treatment.

**Supplementary Information:**

The online version contains supplementary material available at 10.1007/s00592-024-02381-3.

## Background

Type 1 diabetes is a lifelong disease requiring intensive insulin treatment and daily monitoring of blood glucose levels. Despite efforts to maintain the glucose levels as close as possible to the recommended target, the majority of patients do not achieve this goal, leading to an increased risk of acute and chronic complications and adverse effects on quality of life [1].

However, in the last decades there have been many technological advances that have positively impacted the management of type 1 diabetes. First developed in the late 1970s, insulin pumps providing continuous subcutaneous insulin infusion (CSII) have shown to reduce both HbA1c and the rate of hypoglycemic events when compared with multiple daily injections (MDI) of insulin [2]. More recently, continuous glucose monitoring (CGM) with minimally invasive devices has further revolutionized diabetes care, with meaningful improvements in glycemic control, risk of hypoglycemia, and quality of life as compared with self-monitoring of blood glucose (SMBG) [3–7]. CGM devices provide actionable information that is updated every few minutes, including historic and current glucose values, rate of change of glucose, and alarms/alerts for high or low glucose fluctuations. However, they differ from each other by configuration (all-in-one vs. multicomponent devices), type of sensor (transcutaneous vs. fully implantable sensors), visualization tools (handheld receiver and/or smartphone apps), sensor lifetime, data update cycle (real-time vs. intermittently scanned devices), type of glucose alerts (only threshold alerts vs. threshold, predictive, and rate-of-change alerts), possibility of integration with other devices, and other features.

Importantly, the combination of CSII and CGM technologies has resulted in increasing level of automation of insulin delivery in response to sensor glucose readings, ranging from no automation (sensor-augmented pump [SAP] therapy) to algorithm-driven suspension of basal insulin for actual and/or impending hypoglycemia (predictive low glucose management, PLGM), algorithm-driven infusion of basal insulin (hybrid closed loop [HCL] insulin delivery) or algorithm-driven infusion of both basal insulin and correction boluses (advanced hybrid closed loop [AHCL] insulin delivery). In randomized clinical trials, SAP therapy has been associated with significant HbA1c lowering as compared with MDI + SMBG [8], and PLGM and HCL/AHCL systems with reduced hypoglycemia measures and increased time spent within the target glucose range of 70–180 mg/dL (TIR) together with reduced time spent in hypoglycemia, respectively, as compared with SAP [9, 10]. Importantly, HCL/AHCL systems have obtained more favorable psychological outcomes than the comparators in the majority of published trials [11].

Ultimately, several alternative opportunities are nowadays available for the treatment of type 1 diabetes, possibly with different effects on glycemic control and patient-reported outcomes (PROMs). A multicentre, real-world observational study conducted at 22 pediatric diabetes centers in Italy has recently confirmed that patients treated with HCL/AHCL systems achieve the highest TIR and the lowest time spent in hyperglycemia as compared with other therapeutic modalities [12]. Moreover, SAP, PLGM and HCL/AHCL, but not MDI + SMBG, were associated with increased device satisfaction and lower diabetes impact than MDI + CGM as measured by the Diabetes Impact and Device Satisfaction (DIDS) scale.

The current study was designed to evaluate glycemic control and PROMs in a cohort of adult patients with type 1 diabetes using different treatment modalities, including traditional strategies for glucose monitoring and insulin administration and more advanced technological approaches.

## Materials, and methods

This was a multicentre, nationwide, cross-sectional study. Inclusion criteria were: 18 to 60 years of age, being diagnosed with type 1 diabetes for at least six months, being on a MDI- or CSII-based treatment for at least three months, adequate understanding of Italian language. Major exclusion criteria were personal history of psychiatric disease and use of open source automated insulin delivery systems. Candidates were consecutively evaluated for eligibility during their routine medical visit at the diabetes centre and enrolled after giving informed consent.

The study was submitted to local institutional ethics committees (protocol no. 2020 439, approved on March 25, 2021 by the Regional Ethics Committee of Marche, University Hospital “Ospedali Riuniti”, Ancona, Italy, as the coordinating center) and carried out in adherence to Good Clinical Practice, ICH Harmonized Tripartite Guidelines for Good Clinical Practice and Declaration of Helsinki.

### Procedures

Upon obtaining informed consent, researchers collected comprehensive demographic, anamnestic and clinical data, and administered the 11-item DIDS scale. Importantly, collection of demographic data involved socio-economic indicators such as educational attainment (classified as follows: low: lower secondary school or less, medium: upper secondary school, or high: university degree or more), employment status, household annual income (classified as follows: low: <26,000 €, medium: 26,000–54,999 €, or high > 54,999 €) and housing tenure. Time spent doing physical activity was recorded from self-reporting and expressed as hours per week. Number of diabetic ketoacidosis (DKA) and severe hypoglycemia (i.e., requiring third-party assistance) episodes in the past 12 months was collected from both self-reporting and medical records. Finally, for CGM users, the following metrics were obtained from the last 30 days before enrolment: TIR, time spent in hypoglycemia level 1 (< 70 − 54 mg/dL), time spent in hypoglycemia level 2 (< 54 mg/dL), time spent in hyperglycemia level 1 (> 180–250 mg/dL), time spent in hyperglycemia level 2 (> 250 mg/dL), coefficient of variation of glucose (CV), and Glucose Management Indicator (GMI). Devices data sources included Dexcom Clarity (Dexcom, Inc., San Diego, CA, USA), Glooko-Diasend (Glooko, Inc., Mountain View, CA, USA), Medtronic Carelink System (Medtronic, Inc., Minneapolis, MN, USA), and LibreView (Abbott Diabetes Care, Inc., Alameda, CA, USA) platforms.

### Diabetes impact and device satisfaction scale questionnaire

The DIDS questionnaire consists of 11 items, each rated using a 10-point Likert scale, assessing two domains [13]. The first domain comprises seven items measuring satisfaction related to insulin delivery devices, while the second domain includes the remaining four items assessing the impact of diabetes on daily activities, concerns over hypoglycemia, and sleep disturbances. Higher scores in the two domains indicate higher device satisfaction and higher diabetes impact, respectively.

Importantly, the DIDS scale has been recently translated into Italian and validated in a pediatric population [12]. For the purposes of this study, validation assessment was carried out in a subsample of adult participants using CGM devices. Briefly, structural integrity was assessed through confirmatory factor analysis, internal consistency reliability by calculating the Cronbach’s alpha coefficient, and discriminant ability by comparing people with TIR ≥ 75% and TIR < 50% through the Wilcoxon rank-sum test.

### Treatment modalities

For the purposes of our study, the following treatment modalities were compared: MDI + SMBG, MDI + CGM, SAP, PLGM, and HCL/AHCL. All devices were provided by the Italian National Health System with no charge for the patients, regardless of their income, age, or gender.

### Statistical analysis

Continuous variables were expressed as mean ± standard deviation or median (interquartile range), while discrete variables as absolute and percentage frequencies. Normal distribution of continuous variables was assessed through the Shapiro-Wilks test. Demographic, anamnestic and clinical data, treatment satisfaction and impact of diabetes as measured through the DIDS, and CGM-derived glucose metrics were evaluated according to treatment modalities, and the Kruskal-Wallis test was used to compare groups.

The probability of achieving optimal glycemic control (TIR ≥ 70%) with the different treatment modalities was assessed via a logistic regression model; specifically, achieving a TIR ≥ 70% (yes vs. no) was the dependent variable, the therapeutic modality was the explicative factor, and gender, age, disease duration, physical activity, educational attainment, and family income were entered as controlling covariates. Since TIR values were not available for subjects treated with MDI + SMBG, they were not included in this analysis.

Factors associated with device satisfaction and diabetes impact were analysed using quantile regression models. In this analysis, DIDS scores were treated as outcome variables of interest, while treatment modalities were examined as primary factors. To ensure a comprehensive understanding, adjustments were made for a number of variables, including gender, age, disease duration, physical activity, educational attainment, and family income.

## Results

From 2021 to 2022, a total of 428 subjects, 45% males, with median age of 32 years (IQR 23–47) and median diabetes duration of 17 years (IQR 11–25), were recruited in 11 participating centres from all over Italy (Table S1). Main patients’ characteristics are reported in Table 1. Information on treatment modality was available for 427 out of 428 participants. Specifically, 39 (9.1%) subjects were on MDI + SMBG, 155 (36.3%) on MDI + CGM, 99 (23.2%) on SAP therapy, 33 (7.7%) on PLGM, and 101 (23.7%) on HCL/AHCL. All SAP users were using tubeless pumps.

**Table 1:** Main demographic and clinical patients’ characteristics

Characteristics	*n*	
Age, years [median (IQR)]	428	32 (23; 47)
Diabetes duration, years [median (IQR)]	428	17 (11; 25)
Male gender, n (%)	428	193 (45.09)
Glucose Management Indicator, % [mean (SD)]	376	7.1 (0.7)
Therapeutic Strategy, n (%)	427	
MDI + SMBG		39 (9.1)
MDI + CGM		155 (36.3)
SAP		99 (23.2)
PLGM		33 (7.7)
HCL/AHCL		101 (23.7)
Time with glucose level below 54 mg/dL, % [median (IQR)]	386	0.2 (0; 1)
Time with glucose level between 54–69 mg/dL, % [median (IQR)]	386	2 (1; 4)
Time in glucose range 70–180 mg/dL, % [median (IQR)]	386	64 (51.3; 75)
Time with glucose level between 181–250 mg/dL, % [median (IQR)]	386	24 (18; 29.5)
Time with glucose level above 250 mg/dL, % [median (IQR)]	386	7 (3; 14)
Subjects with at least one episode of hypoglycemia in the previous year, n, %	426	33 (7.7)
Subjects with at least one episode of DKA in the previous year, n, %	427	6 (1.4)
Frequency of SMBG, tests/day [median (IQR)]	427	2 (0; 4)
Physical activity, hours / week [median (IQR)]	427	2 (0; 4)
Geografical area, n (%)	428	
North		126 (29.4)
Centre		63 (14.7)
Sud		239 (55.8)
Educational level, n (%)	412	
Low		39 (9.4)
Medium		280 (67.8)
High		93 (22.8)
Family gross annual income, n (%)	363	
Low		148 (40.8)
Medium		167 (46.0)
High		48 (13.2)

IQR: interquartile range; SD: standard deviation; MDI: multiple daily injections of insulin; SMBG: self-monitoring of blood glucose; CGM: continuous glucose monitoring; SAP: sensor-augmented pump; PLGM: predictive low glucose management; HCL: hybrid closed loop; AHCL: advanced hybrid closed loop

**Fig. 1:**
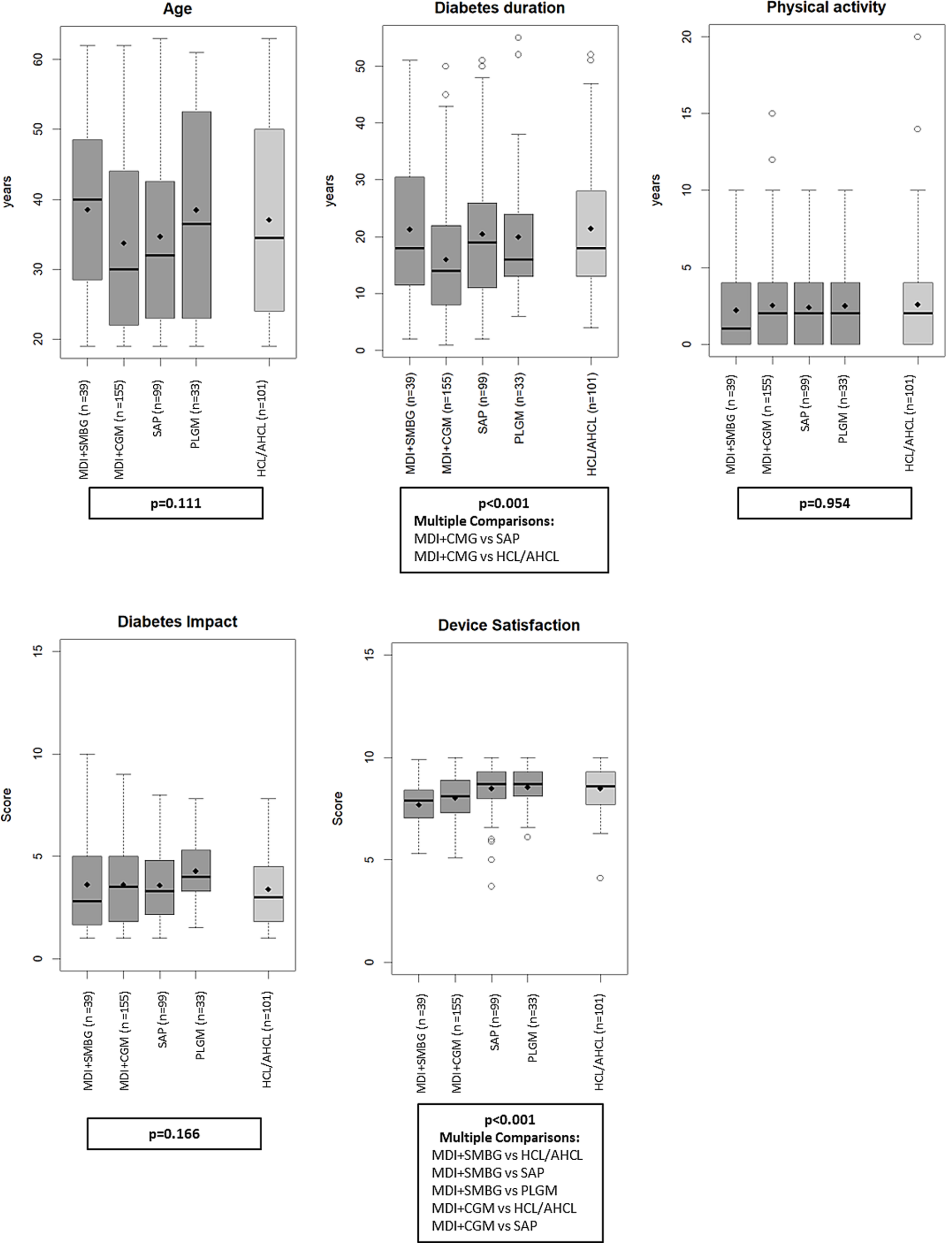
Subjects’ demographic and clinical characteristics by treatment modalities. MDI: multiple daily injections of insulin; SMBG: self-monitoring of blood glucose; CGM: continuous glucose monitoring; PLGM: predictive low glucose management; HCL: hybrid closed loop; AHCL: advanced hybrid closed loop

Figure 1 and Table S2 show the demographic and clinical characteristics of the subjects by treatment modalities. No statistically significant differences in age, physical activity, and diabetes impact were found among treatment modalities. Patients treated with SAP and HCL/AHCL had a significantly longer diabetes duration [19 (11–26) years and 18 (13–28) years, respectively] than those treated with MDI + CGM [14 (8–22) years], and reported higher device satisfaction vs. both MDI + SMBG and MDI + CGM. Patients treated with PLGM also exhibited a significantly higher device satisfaction than MDI + SMBG.

**Fig. 2:**
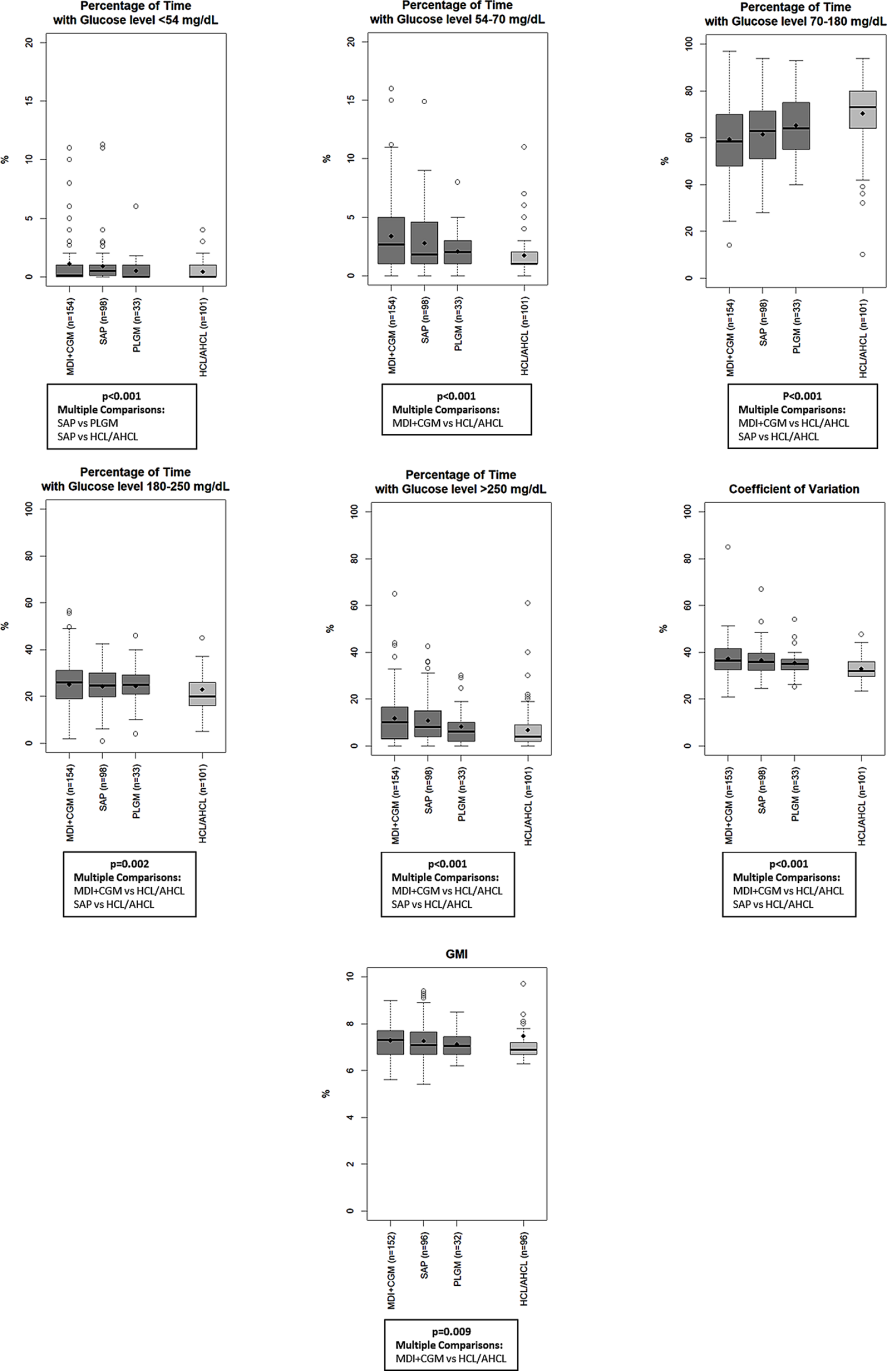
Glucose metrics by treatment modalities. MDI: multiple daily injections of insulin; SMBG: self-monitoring of blood glucose; CGM: continuous glucose monitoring; PLGM: predictive low glucose management; HCL: hybrid closed loop; AHCL: advanced hybrid closed loop

Subjects treated with HCL/AHCL exhibited significantly higher TIR [73% (64–80)] and significantly lower time spent in hypoglycemia level 1 [1% (1–2)], time spent in hyperglycemia [20% (16–26)], CV [32% (29.7–36)] and GMI [6.9% (6.7–7.2)] compared to patients treated with MDI + CGM, and significantly higher TIR and significantly lower time spent in hypoglycemia level 2 [0% (0–1)], time spent in hyperglycemia, and CV compared to SAP therapy (Fig. 2, Table S2). Significant reduction in hypoglycemia level 2 was also found with PLGM as compared with SAP therapy. Number of self-reported episodes of DKA and severe hypoglycemia was generally low (Table S3).

The use of HCL/AHCL systems increased the probability of being at TIR target (≥ 70%) by 5.1 times compared to MDI + CGM (*p* < 0.001), independently from demographic, clinical and anamnestic variables. Furthermore, completing a “high” level of education increased the probability of being at TIR ≥ 70% by 3.5 times compared to a low level (*p* = 0.036). The probability of having TIR ≥ 70% also increased by 2% and 9% for each year of age and each hour of physical activity added, respectively (Table 2).

**Table 2:** Variables associated to optimal metabolic control (TIR ≥ 70%, *n* = 143)

	OR	95%CI	*p*
Gender: female vs. male	0.76	0.45; 1.26	0.282
Age (years)	1.02	1.01; 1.06	**0.004**
Diabetes duration (years)	0.99	0.96; 1.02	0.410
Therapy: SAP vs. MDI + CGM	1.25	0.66; 2.38	0.498
Therapy: PLGM vs. MDI + CGM	1.27	0.48; 3.18	0.615
Therapy: HCL/AHCL vs. MDI + CGM	5.06	2.68; 9.79	**< 0.001**
Physical activity (hours/week)	1.09	1.01; 1.19	**0.035**
Education level: medium vs. low	1.98	0.74; 6.02	0.196
Education level: high vs. low	3.46	1.17; 11.39	**0.031**
Family annual income: medium vs. low	0.78	0.45; 1.36	0.383
Family annual income: high vs. Low	0.91	0.4; 2.05	0.823

TIR: time in range; SAP: sensor-augmented pump; MDI: multiple daily injections of insulin; SMBG: self-monitoring of blood glucose; CGM: continuous glucose monitoring; PLGM: predictive low glucose management; HCL: hybrid closed loop; AHCL: advanced hybrid closed loop; OR: Odds Ratio; 95% CI: 95% Confidence Interval. Statistically significant results are in bold

Table 3 reports the predictors of device satisfaction and diabetes impact as assessed with quantile regression analysis. Compared to MDI + CGM, SAP and HCL/AHCL treatments were significantly and independently associated with higher device satisfaction, MDI + SMBG with lower device satisfaction, and PLGM with higher diabetes impact. With regards to demographic and socio-economic characteristics, higher diabetes duration was associated with a higher device satisfaction, while increasing age and having a “medium” vs. “low” family income were both associated with lower diabetes impact.

**Table 3:** Variables associated with DIDS domains. Results of quantile regression analysis

	Device Satisfaction		Diabetes Impact	
Variables	B	95%CI	b	95%CI
Gender: female vs. male	0.03	-0.12; 0.34	0.36	-0.12; 0.81
Age (years)	-0.01	-0.02; 0.01	**-0.02**	-0.04; -0.01
Diabetes duration (years)	**0.02**	0.01; 0.04	-0.01	-0.04; 0.01
Therapy: MDI + SMBG vs. MDI + CGM	**-0.71**	-1.2; -0.01	0.42	-0.5; 2.08
Therapy: SAP vs. MDI + CGM	**0.30**	0.02; 0.59	0.16	-0.45; 0.88
Therapy: PLGM vs. MDI + CGM	0.29	-0.1; 0.65	**1.18**	0.03; 1.71
Therapy: HCL/AHCL vs. MDI + CGM	0.31	-0.08; 0.62	0	-0.9; 0.58
Physical activity (hours/week)	-0.03	-0.06; 0.03	0.04	-0.01; 0.1
Education level: medium vs. low	-0.17	-0.49; 0.26	-0.23	-0.97; 0.34
Education level: high vs. low	-0.11	-0.51; 0.36	0.13	-0.95; 0.79
Family annual income: medium vs. low	0.05	-0.28; 0.29	**-0.74**	-1.11; -0.21
Family annual income: high vs. low	0.41	-0.04; 0.72	0.11	-0.38; 0.81

b: quantile regression coefficient; 95%CI: 95% Confidence Interval; MDI: multiple daily injections of insulin; SMBG: self-monitoring of blood glucose; CGM: continuous glucose monitoring; PLGM: predictive low glucose management; HCL: hybrid closed loop; AHCL: advanced hybrid closed loop. Statistically significant results are in bold

The validation assessment of the Italian version of the DIDS scale was conducted in a subsample of 389 CGM users, 45% male, with a median age of 32 years (23–47), and showed moderate level of structural integrity (Table S4, Figure S1) and good internal consistency (Tables S5).

In detail, internal consistency estimates were 0.71 (95% CI: 0.66; 0.75) and 0.74 (95% CI: 0.70; 0.78) for Device Satisfaction and Diabetes Impact domains, respectively. When evaluating the contribution of each single item to overall internal consistency, the Cronbach’s a coefficient ranged from 0.63 to 0.74. The discriminant validity assessment was carried out comparing 75 participants with TIR < 50% with 100 participants with TIR ≥ 75%. Significant differences in both DIDS domains were observed, with higher median device satisfaction and lower diabetes impact being reported for participants with TIR ≥ 75% (Figure S2).

## Discussion

In the last few years, there have been many technological advances in glucose monitoring and insulin delivery, which have resulted in new opportunities for the treatment of type 1 diabetes.

The results of our study show that adult HCL/AHCL users with type 1 diabetes achieve the highest TIR, the lowest time spent in hyperglycemia, and the lowest time spent in hypoglycemia compared to other CGM-enhanced treatment modalities, with statistically significant differences being reported vs. MDI and SAP therapies. What’s more, HCL/AHCL users exhibited median TIR values that met the recommended target of > 70% for non-fragile non-pregnant adults with type 1 diabetes [14] with negligible time spent in hypoglycemia, and use of HCL/AHCL systems was identified as the single best predictor of achieving optimal metabolic control.

Randomized clinical trials and other observational studies have already shown the superiority of such systems in providing favourable glycemic outcomes as compared with other treatment modalities [10, 15], however our report is the first in the literature focusing on the Italian scenario and evaluating also the socioeconomic status of users, with more than 400 adult participants enrolled in 11 diabetes centres from all over the country.

In our analysis, PLGM was associated with lower time spent < 54 mg/dL than SAP therapy. Reduction of hypoglycemia measures with PLGM systems has also been replicated in randomized clinical trials and real-world studies [9, 16]. In line with these findings, international guidelines recommend use of integrated CGM and insulin pump systems proving automated insulin suspension/dosing over non-integrated systems in persons with type 1 diabetes [17, 18].

Time spent in hypoglycemia was numerically similar between HCL/ACHL and PLGM users. However, this is not surprising; in fact, according to the results of different RCT and real-world studies, superiority of HCL/AHLC systems vs. PLGM for hypoglycemia reduction has yet to be proven with certainty [19].

Interestingly, occurrence of severe hypoglycemic episodes was infrequent with any treatment modality, the proportion of patients experiencing at least one episode being lower than that reported in the Study of Adults’ GlycEmia in T1DM (SAGE), therefore confirming a high level of commitment in the management of hypoglycemia among the Italian patients [1, 20].

Use of technological devices for glucose monitoring and/or insulin administration was generally associated with higher device satisfaction without increased disease burden compared to the traditional approach, except for PLGM. In recent years, the assessment of PROMs, including quality of life and satisfaction with treatments and technologies, has progressively emerged as a critical factor for successful management of type 1 diabetes [11]. Indeed, patient satisfaction has been linked with persistent use of devices and improved glycemic control [21, 22]. For the purposes of our analysis, HCL and AHCL users were pooled together, however there is evidence in the literature that users’ acceptance is increased with AHCL as compared with earlier systems, maybe due to frequent alarms and need for calibration by fingerstick glucose to maintain the Auto-Mode with the latters [23, 24].

With regards to both glycemic outcomes and patient satisfaction, the results of our adult cohort are in line with those of the recently published pediatric study [12], therefore confirming that diabetes devices are beneficial in the whole spectrum of patients with type 1 diabetes, and HCL/AHCL systems represent nowadays the gold standard of insulin replacement treatment [25]. Nevertheless, use of technological devices is still limited in Italy, with only 40.8% and 24% of patients with type 1 diabetes being on CGM or an insulin pump, respectively, the levels of uptake being even lower among adults and in southern regions [26]. Inadequate number of professionals in the diabetes team, need for high-level training of both healthcare professionals and patients, insufficient allocation of economic resources, and heterogeneous reimbursement policies are well-known barriers for a wider spread of diabetes devices [27, 28]. We hope that our research may convince both the healthcare professionals and the payers of the irreplaceable role of technology for the management of type 1 diabetes mellitus.

Interestingly, completing a “high” level of education was independently associated with reaching ≥ 70% of TIR among CGM users. This is not surprisingly when one recalls that self-management of type 1 diabetes requires numerical skills and simultaneous consideration of multiple variables (e.g., deviation from target glucose, glucose trend, carbohydrate intake, insulin sensitivity factor, insulin on board, etc.) before making treatment decisions [29, 30]. However, glucose-driven automated insulin delivery in PLGM and HCL/AHCL systems may compensate some patients’ deficiencies [31, 32], and therefore “democratize” insulin treatment.

Increasing age and time spent for physical activity were similarly linked to optimal metabolic control. In the literature, conflicting results in terms of overall glycemic control have been reported with exercise in individuals with type 1 diabetes, with some studies demonstrating benefits [33–35] and others no improvement in HbA1c following aerobic or resistance training [36, 37]. To achieve enhanced glycemic control while avoiding hypoglycemia, a skillful balance of insulin dosing and food intake is required before, during, and after exercise [38]. Technological advances may help accomplish these tasks with less effort [39].

The independent association of diabetes duration with device satisfaction is also intriguing, in agreement with recent research showing better technology utilization in patients with long-standing disease [40].

The major strengths of our study are the large cohort of participants, the consecutive enrolment, and the great number of outcomes and characteristics that were considered. However, there are some limitations that should be acknowledged. First of all, the cross-sectional design formally prevents causal and temporal inferences between treatments modalities and their glycemic and psychosocial correlates. However, randomized clinical trials and their meta-analyses have extensively clarified that HCL/AHCL systems lead to unprecedented improvements in both aspects of diabetes management [10, 11]. In this scenario, our research shows that treatment goals are achieved also in the Italian real-world setting, therefore providing a reassuring insight to both healthcare professionals and payers. Second, participants on MDI + SMBG were few in number as compared with other groups. In this regard, it has to be considered that our research was conducted in centres with high levels of uptake of diabetes technologies and high expertise in this field, where technology naïve patients are undoubtedly a minority. Third, as laboratory-measured HbA1c levels were not available for the majority of participants, these data were not analyzed. Finally, we used a single tool for the assessment of PROMs, and some important aspects including fear of hypoglycaemia, sleep quality, and diabetes distress were not evaluated. However, the DIDS scale is a short and easy-to-administer tool that is adequate for use across all insulin delivery devices, and has shown robust psychometric properties in individuals with type 1 diabetes [13].

## Conclusion

In adults with type 1 diabetes from different areas of Italy, real-life use of advanced technologies for glucose monitoring and/or insulin delivery, particularly HCL/AHCL systems, is associated with improved glucose metrics and device satisfaction. In this population, education attainment, but not family income, may impact on glycemic outcomes. While a definitive cure for type 1 diabetes is not yet achievable, it is crucial to increase the uptake of the most efficacious treatment options to everyone who can benefit from it.

## Electronic supplementary material

Below is the link to the electronic supplementary material.

Supplementary Material 1

## Data Availability

Aggregated data might be made available upon reasonable request via email to the corresponding author.

## References

[1] Renard E, Ikegami H, Daher Vianna AG, Pozzilli P, Brette S, Bosnyak Z, Lauand F, Peters A, Pilorget V, Jurišić-Eržen D, Kesavadev J, Seufert J, Wilmot EG (2021) The SAGE study: global observational analysis of glycaemic control, hypoglycaemia and diabetes management in T1DM. Diabetes Metab Res Rev 37(7):e3430. 10.1002/dmrr.3430Epub 2021 Mar 2. PMID: 33369842; PMCID: PMC8518876 33369842 10.1002/dmrr.3430 PMC8518876

[2] Pickup JC, Sutton AJ (2008) Severe hypoglycaemia and glycaemic control in type 1 diabetes: meta-analysis of multiple daily insulin injections compared with continuous subcutaneous insulin infusion. Diabet Med 25:765–774 18644063 10.1111/j.1464-5491.2008.02486.x

[3] Dicembrini I, Cosentino C, Monami M, Mannucci E, Pala L (2021) Effects of real-time continuous glucose monitoring in type 1 diabetes: a meta-analysis of randomized controlled trials. Acta Diabetol 58:401–410 32789691 10.1007/s00592-020-01589-3

[4] Castellana M, Parisi C, Di Molfetta S, Di Gioia L, Natalicchio A, Perrini S, Cignarelli A, Laviola L, Giorgino F (2020) Efficacy and safety of flash glucose monitoring in patients with type 1 and type 2 diabetes: a systematic review and meta-analysis. BMJ Open Diabetes Res Care 8(1):e001092. 10.1136/bmjdrc-2019-001092PMID: 32487593; PMCID: PMC7265013 32487593 10.1136/bmjdrc-2019-001092 PMC7265013

[5] Irace C, Cutruzzolà A, Nuzzi A, Assaloni R, Brunato B, Pitocco D, Tartaglione L, Di Molfetta S, Cignarelli A, Laviola L, Citro G, Lovati E, Gnasso A, Tweden KS, Kaufman FR (2020) Clinical use of a 180-day implantable glucose sensor improves glycated haemoglobin and time in range in patients with type 1 diabetes. Diabetes Obes Metab 22(7):1056–1061. 10.1111/dom.13993Epub 2020 Feb 27. PMID: 32037699; PMCID: PMC7317779 32037699 10.1111/dom.13993 PMC7317779

[6] Charleer S, Mathieu C, Nobels F, De Block C, Radermecker RP, Hermans MP, Taes Y, Vercammen C, T’Sjoen G, Crenier L, Fieuws S, Keymeulen B, Gillard P (2018) RESCUE Trial Investigators. Effect of Continuous Glucose Monitoring on Glycemic Control, Acute Admissions, and Quality of Life: A Real-World Study. J Clin Endocrinol Metab. 103(3):1224–1232. 10.1210/jc.2017-02498. PMID: 29342264 10.1210/jc.2017-02498 29342264

[7] Ang E, Lee ZX, Moore S, Nana M (2020) Flash glucose monitoring (FGM): a clinical review on glycaemic outcomes and impact on quality of life. J Diabetes Complications 34:107559 32089428 10.1016/j.jdiacomp.2020.107559

[8] Bergenstal RM, Tamborlane WV, Ahmann A, Buse JB, Dailey G, Davis SN, Joyce C, Perkins BA, Welsh JB, Willi SM, Wood MA, STAR 3 Study Group (2011) Sensor-augmented pump therapy for A1C reduction (STAR 3) study: results from the 6-month continuation phase. Diabetes Care 34(11):2403–2405. 10.2337/dc11-1248Epub 2011 Sep 20. PMID: 21933908; PMCID: PMC3198292 21933908 10.2337/dc11-1248 PMC3198292

[9] Abraham MB, Nicholas JA, Smith GJ, Fairchild JM, King BR, Ambler GR, Cameron FJ, Davis EA, Jones TW, PLGM Study Group (2018) Reduction in Hypoglycemia with the predictive low-glucose management system: a long-term Randomized Controlled Trial in adolescents with type 1 diabetes. Diabetes Care 41(2):303–310. 10.2337/dc17-1604Epub 2017 Nov 30. PMID: 29191844 29191844 10.2337/dc17-1604

[10] Biester T, Tauschmann M, Chobot A, Kordonouri O, Danne T, Kapellen T, Dovc K (2022) The automated pancreas: a review of technologies and clinical practice. Diabetes Obes Metab 24(Suppl 1):43–57. 10.1111/dom.14576Epub 2021 Nov 14. PMID: 34658126 34658126 10.1111/dom.14576

[11] Franceschi R, Mozzillo E, Di Candia F, Maines E, Leonardi L, Girardi M, Fedi L, Rosanio FM, Marcovecchio ML (2023) A systematic review on the impact of commercially available hybrid closed loop systems on psychological outcomes in youths with type 1 diabetes and their parents. Diabet Med 40(9):e15099. 10.1111/dme.15099Epub 2023 Apr 17. PMID: 37029751 37029751 10.1111/dme.15099

[12] Cherubini V, Fargalli A, Arnaldi C, Bassi M, Bonfanti R, Patrizia Bracciolini G, Cardella F, Dal Bo S, Delvecchio M, Di Candia F, Franceschi R, Maria Galassi S, Gallo F, Graziani V, Iannilli A, Mameli C, Marigliano M, Minuto N, Monti S, Mozzillo E, Pascarella F, Predieri B, Rabbone I, Roppolo R, Schiaffini R, Tiberi V, Tinti D, Toni S, Scaramuzza A, Vestrucci B, Gesuita R (2024) Glucometrics and device satisfaction in children and adolescents with type 1 diabetes using different treatment modalities: a multicenter real-world observational study. Diabetes Res Clin Pract 210:111621. 10.1016/j.diabres.2024.111621Epub 2024 Mar 16. PMID: 38499182 38499182 10.1016/j.diabres.2024.111621

[13] Manning ML, Singh H, Stoner K, Habif S (2020) The development and psychometric validation of the diabetes impact and device satisfaction scale for individuals with type 1 diabetes. J Diabetes Sci Technol 14(2):309–317 32028790 10.1177/1932296819897976 PMC7196859

[14] Battelino T, Danne T, Bergenstal RM, Amiel SA, Beck R, Biester T, Bosi E, Buckingham BA, Cefalu WT, Close KL, Cobelli C, Dassau E, DeVries JH, Donaghue KC, Dovc K, Doyle FJ 3rd, Garg S, Grunberger G, Heller S, Heinemann L, Hirsch IB, Hovorka R, Jia W, Kordonouri O, Kovatchev B, Kowalski A, Laffel L, Levine B, Mayorov A, Mathieu C, Murphy HR, Nimri R, Nørgaard K, Parkin CG, Renard E, Rodbard D, Saboo B, Schatz D, Stoner K, Urakami T, Weinzimer SA, Phillip M (2019) Clinical targets for continuous glucose Monitoring Data Interpretation: recommendations from the International Consensus on Time in Range. Diabetes Care 42(8):1593–1603. 10.2337/dci19-0028Epub 2019 Jun 8. PMID: 31177185; PMCID: PMC6973648 10.2337/dci19-0028 PMC6973648 31177185

[15] Beato-Víbora PI, Gallego-Gamero F, Ambrojo-López A (2021) Real-world outcomes with different technology modalities in type 1 diabetes. Nutr Metab Cardiovasc Dis. 31(6):1845–1850. 10.1016/j.numecd.2021.02.028. Epub 2021 Mar 3. PMID: 33838993 10.1016/j.numecd.2021.02.028 33838993

[16] Müller L, Habif S, Leas S, Aronoff-Spencer E (2019) Reducing hypoglycemia in the Real World: a retrospective analysis of predictive low-glucose Suspend Technology in an ambulatory insulin-dependent cohort. Diabetes Technol Ther 21(9):478–484. 10.1089/dia.2019.0190Epub 2019 Aug 1. PMID: 31329468; PMCID: PMC6708266 31329468 10.1089/dia.2019.0190 PMC6708266

[17] Grunberger G, Sherr J, Allende M, Blevins T, Bode B, Handelsman Y, Hellman R, Lajara R, Roberts VL, Rodbard D, Stec C, Unger J (2021) American Association of Clinical Endocrinology Clinical Practice Guideline: The Use of Advanced Technology in the Management of Persons With Diabetes Mellitus. Endocr Pract. 27(6):505–537. 10.1016/j.eprac.2021.04.008. PMID: 34116789 10.1016/j.eprac.2021.04.008 34116789

[18] Associazione dei Medici Diabetologi (AMD), della Società Italiana di Diabetologia (SID) e della Società Italiana di Endocrinologia e Diabetologia Pediatrica (SIEDP). Linea Guida della Associazione dei Medici Diabetologi (AMD), della Società Italiana di Diabetologia (SID) e della Società Italiana di Endocrinologia e Diabetologia Pediatrica (SIEDP) La terapia del diabete mellito di tipo 1. Accessed on 11 (2024) https://www.iss.it/-/snlg-diabete-mellito-tipo1

[19] Rossi A, Montefusco L, Reseghetti E, Pastore IF, Rossi G, Usuelli V, Loretelli C, Boci D, Ben Nasr M, D’Addio F, Bucciarelli L, Argenti S, Morpurgo P, Lunati ME, Fiorina P (2023) Daytime hypoglycemic episodes during the use of an advanced hybrid closed loop system. Diabetes Res Clin Pract. 206:111011. 10.1016/j.diabres.2023.111011. Epub 2023 Nov 11. PMID: 37956944 10.1016/j.diabres.2023.111011 37956944

[20] Bruttomesso D, Irace C, Pozzilli P, SAGE study group (2023) A sub-analysis of the SAGE study in Italy indicates good glycemic control in type 1 diabetes. Nutr Metab Cardiovasc Dis 33(3):631–639 Epub 2022 Nov 17. PMID: 36670006 36670006 10.1016/j.numecd.2022.11.008

[21] Diabetes Research in Children Network (DirecNet) Study Group (2005) Youth and parent satisfaction with clinical use of the GlucoWatch G2 biographer in the management of pediatric type 1 diabetes. Diabetes Care 28(8):1929–1935. 10.2337/diacare.28.8.1929PMID: 16043734; PMCID: PMC1414784 16043734 10.2337/diacare.28.8.1929 PMC1414784

[22] Tansey M, Laffel L, Cheng J, Beck R, Coffey J, Huang E, Kollman C, Lawrence J, Lee J, Ruedy K, Tamborlane W, Wysocki T, Xing D, Juvenile Diabetes Research Foundation Continuous Glucose Monitoring Study Group (2011). Satisfaction with continuous glucose monitoring in adults and youths with Type 1 diabetes. Diabet Med. 28(9):1118-22. 10.1111/j.1464-5491.2011.03368.x. PMID: 21692844 10.1111/j.1464-5491.2011.03368.x 21692844

[23] Lal RA, Basina M, Maahs DM, Hood K, Buckingham B, Wilson DM (2019) One year clinical experience of the First Commercial Hybrid closed-Loop System. Diabetes Care 42(12):2190–2196. 10.2337/dc19-0855Epub 2019 Sep 23. PMID: 31548247; PMCID: PMC6868462 31548247 10.2337/dc19-0855 PMC6868462

[24] Yuan CY, Kong YW, Amoore T, Brown K, Grosman B, Jenkins A, Jones H, Kurtz N, Lee MH, MacIsaac R, Netzer E, Paldus B, Robinson L, Roy A, Sims CM, Trawley S, Vogrin S, O’Neal DN (2024) Improved Satisfaction While Maintaining Safety and High Time in Range (TIR) With a Medtronic Investigational Enhanced Advanced Hybrid Closed-Loop (e-AHCL) System. Diabetes Care. 47(4):747–755. 10.2337/dc23-2217. PMID: 38381515 10.2337/dc23-2217 38381515

[25] Phillip M, Nimri R, Bergenstal RM, Barnard-Kelly K, Danne T, Hovorka R, Kovatchev BP, Messer LH, Parkin CG, Ambler-Osborn L, Amiel SA, Bally L, Beck RW, Biester S, Biester T, Blanchette JE, Bosi E, Boughton CK, Breton MD, Brown SA, Buckingham BA, Cai A, Carlson AL, Castle JR, Choudhary P, Close KL, Cobelli C, Criego AB, Davis E, de Beaufort C, de Bock MI, DeSalvo DJ, DeVries JH, Dovc K, Doyle FJ, Ekhlaspour L, Shvalb NF, Forlenza GP, Gallen G, Garg SK, Gershenoff DC, Gonder-Frederick LA, Haidar A, Hartnell S, Heinemann L, Heller S, Hirsch IB, Hood KK, Isaacs D, Klonoff DC, Kordonouri O, Kowalski A, Laffel L, Lawton J, Lal RA, Leelarathna L, Maahs DM, Murphy HR, Nørgaard K, O’Neal D, Oser S, Oser T, Renard E, Riddell MC, Rodbard D, Russell SJ, Schatz DA, Shah VN, Sherr JL, Simonson GD, Wadwa RP, Ward C, Weinzimer SA, Wilmot EG, Battelino T (2023) Consensus recommendations for the Use of Automated insulin Delivery technologies in clinical practice. Endocr Rev 44(2):254–280. 10.1210/endrev/bnac022PMID: 36066457; PMCID: PMC9985411 36066457 10.1210/endrev/bnac022 PMC9985411

[26] Pitocco D, Laurenzi A, Tomaselli L, Assaloni R, Consoli A, Di Bartolo P, Guardasole V, Lombardo F, Maffeis C, Rossi A, Gesuita R, Di Molfetta S, Rigamonti A, Scaramuzza A, Irace C, Cherubini V, Working group of Diabetes and Technology AMD-SID-SIEDP (2022) Health care organization and use of technological devices in people with diabetes in Italy: results from a survey of the Working Group on Diabetes and Technology. Nutr Metab Cardiovasc Dis 32(10):2392–2398 Epub 2022 Jul 16. PMID: 35970683 35970683 10.1016/j.numecd.2022.07.003

[27] Pauley ME, Berget C, Messer LH, Forlenza GP (2021) Barriers to Uptake of Insulin Technologies and Novel solutions. Med Devices (Auckl) 14:339–354. 10.2147/MDER.S312858PMID: 34803408; PMCID: PMC8594891 34803408 10.2147/MDER.S312858 PMC8594891

[28] Sumnik Z, Szypowska A, Iotova V, Bratina N, Cherubini V, Forsander G, Jali S, Raposo JF, Stipančic G, Vazeou A, Veeze H, Lange K, SWEET study group (2019). Persistent heterogeneity in diabetes technology reimbursement for children with type 1 diabetes: The SWEET perspective. Pediatr Diabetes. 20(4):434–443. 10.1111/pedi.12833. Epub 2019 Apr 11. PMID: 30773756 10.1111/pedi.12833 30773756

[29] Marden S, Thomas PW, Sheppard ZA, Knott J, Lueddeke J, Kerr D (2012) Poor numeracy skills are associated with glycaemic control in Type 1 diabetes. Diabet Med.29(5):662-9. 10.1111/j.1464-5491.2011.03466.x. PMID: 21978203 10.1111/j.1464-5491.2011.03466.x 21978203

[30] Kerr D (2010) Poor numeracy: the elephant in the diabetes technology room. J Diabetes Sci Technol 4(6):1284–1287. 10.1177/193229681000400601PMID: 21129322; PMCID: PMC3005037 21129322 10.1177/193229681000400601 PMC3005037

[31] Abraham MB, de Bock M, Paramalingam N, O’Grady MJ, Ly TT, George C, Roy A, Spital G, Karula S, Heels K, Gebert R, Fairchild JM, King BR, Ambler GR, Cameron F, Davis EA, Jones TW (2016) Prevention of Insulin-Induced Hypoglycemia in Type 1 Diabetes with Predictive Low Glucose Management System. Diabetes Technol Ther. 18(7):436 – 43. 10.1089/dia.2015.0364. Epub 2016 May 5. PMID: 27148807 10.1089/dia.2015.0364 27148807

[32] Petrovski G, Campbell J, Pasha M, Day E, Hussain K, Khalifa A, van den Heuvel T (2023) Simplified meal announcement Versus Precise Carbohydrate counting in adolescents with type 1 diabetes using the MiniMed 780G Advanced Hybrid Closed Loop System: a randomized controlled trial comparing glucose control. Diabetes Care 46(3):544–550. 10.2337/dc22-1692PMID: 36598841; PMCID: PMC10148675 36598841 10.2337/dc22-1692 PMC10148675

[33] Cuenca-Garcia M, Jago R, Shield JP, Burren CP (2012) How does physical activity and fitness influence glycaemic control in young people with type 1 diabetes? Diabet Med 10.1111/j.1464-5491.2012.03740.x 22803800

[34] Aouadi R, Khalifa R, Aouidet A et al (2011) Aerobic training programs and glycemic control in diabetic children in relation to exercise frequency. J Sports Med Phys Fit 51:393–400 21904277

[35] Schweiger B, Klingensmith G, Snell-Bergeon JK (2010) Physical activity in adolescent females with type 1 diabetes. Int J Pediatr 2010:328318 20652080 10.1155/2010/328318 PMC2905719

[36] Ramalho AC, de Lourdes Lima M, Nunes F et al (2006) The effect of resistance versus aerobic training on metabolic control in patients with type-1 diabetes mellitus. Diabetes Res Clin Pract 72:271–276 16406128 10.1016/j.diabres.2005.11.011

[37] Aman J, Skinner TC, de Beaufort CE, Swift PG, Aanstoot HJ, Cameron F (2009) Associations between physical activity, sedentary behavior, and glycemic control in a large cohort of adolescents with type 1 diabetes: the Hvidoere Study Group on Childhood Diabetes. Pediatr Diabetes 10:234–239. 10.1111/j.399-5448.2008.00495.x 19140898 10.1111/j.1399-5448.2008.00495.x

[38] Colberg SR, Laan R, Dassau E, Kerr D (2015) Physical activity and type 1 diabetes: time for a rewire? J Diabetes Sci Technol 9(3):609–618 doi: 10.1177/1932296814566231. Epub 2015 Jan 6. PMID: 25568144; PMCID: PMC4604550 25568144 10.1177/1932296814566231 PMC4604550

[39] Tagougui S, Taleb N, Rabasa-Lhoret R (2019) The benefits and limits of Technological advances in glucose management around physical activity in patients type 1 diabetes. Front Endocrinol (Lausanne) 9:818. 10.3389/fendo.2018.00818PMID: 30713524; PMCID: PMC6346637 30713524 10.3389/fendo.2018.00818 PMC6346637

[40] González-Vidal T, Rivas-Otero D, Agüeria-Cabal P, Ramos-Ruiz G, Delgado E, Menéndez-Torre E (2024) Continuous glucose monitoring alarms in adults with type 1 diabetes: user characteristics and the Impact of Hypoglycemia and hyperglycemia alarm thresholds on Glycemic Control. Diabetes Technol Ther 26(5):313–323 Epub 2024 Mar 8. PMID: 38156962 38156962 10.1089/dia.2023.0460

