# Supplementary material for: Glucose metrics and device satisfaction in adults with type 1 diabetes using different treatment modalities: a multicenter, real-world observational study
Source: Acta Diabetol. 2024 Oct 10;62(4):563–73. doi: 10.1007/s00592-024-02381-3 (PMC12055876; doi:10.1007/s00592-024-02381-3)
Supplement: Supplementary file 1 — Supplementary Material 1 [file 592_2024_2381_MOESM1_ESM.docx]

**Table S1. Geographical distribution of adults with type 1 diabetes by diabetes centres.**

| **Centre** | **n** | **%** |
| --- | --- | --- |
| Caserta | 1 | 0.23 |
| Catanzaro | 104 | 24.30 |
| Genova | 57 | 13.32 |
| Milano Sacco | 59 | 13.78 |
| Modena | 10 | 2.33 |
| Napoli Federico II | 17 | 3.97 |
| Palermo | 28 | 6.54 |
| Policlinico Bari | 68 | 15.89 |
| Rome | 39 | 9.11 |
| Sardegna | 21 | 4.91 |
| Viterbo | 24 | 5.61 |
| **Total** | **428** |  |

**Table S2. Metabolic control, glucose metrics, treatment satisfaction and diabetes impact according to therapeutic strategies.**

| **Median (IQR)** | MDI+SMBG |  | MDI+CGM | SAP | PLGM | HCL/AHCL | p |
| --- | --- | --- | --- | --- | --- | --- | --- |
| Glucose Management Indicator (%) | - |  | 7.3 (6.7 - 7.7) | 7.1 (6.7 - 7.6) | 7 (6.7 - 7.4) | 6.9 (6.7 - 7.2) | 0.009 |
| Time below 54mg/dL (%) | - |  | 0.1 (0 - 1) | 0.5 (0.1 - 1) | 0 (0 - 1) | 0 (0 - 1) | p<0.001 |
| Time below 70mg/dL (%) | - |  | 2.6 (1 - 5) | 1.8 (1 - 4.4) | 2 (1 - 3) | 1 (1 - 2) | p<0.001 |
| Time in range 70-180mg/dL (%) | - |  | 58.5 (48 - 70) | 62.8 (51.5 - 71.2) | 64 (55 - 75) | 73 (64 - 80) | p<0.001 |
| Time above 180 mg/dL (%) | - |  | 26 (19 - 31) | 24.6 (19.9 - 30.1) | 25 (21 - 29.2) | 20 (16 - 26) | 0.002 |
| Time above 250mg/dL (%) | - |  | 10 (3 - 16.6) | 8.1 (3.9 - 15) | 6 (2 - 10) | 4 (2 - 9) | p<0.001 |
| Coefficient of variation (%) | - |  | 36.4 (32.6 - 41.5) | 35.8 (32.3 - 39.6) | 35 (32.4 - 37) | 32 (29.7 - 36) | p<0.001 |
| Satisfaction | 7.9 (7.0 - 8.4) |  | 8.1 (7.3 - 8.9) | 8.7 (8 - 9.3) | 8.7 (8.1 - 9.3) | 8.6 (7.7 - 9.3) | p<0.001 |
| Impact | 2.8 (1.6 – 5.0) |  | 3.5 (1.8 - 5) | 3.3 (2.1 - 4.8) | 4 (3.3 - 5.3) | 3 (1.8 - 4.5) | 0.166 |
| Age (years) | 40 (29 - 49) |  | 30 (22 - 44) | 32 (23 - 42.5) | 36.5 (23 - 52.2) | 34.5 (24.5 - 50) | 0.111 |
| Diabetes duration (years) | 18 (12 - 31) |  | 14 (8 – 22) | 19 (11 – 26) | 16 (13 – 24) | 18 (13 – 28) | <0.001 |
| Physical activity (hours/week) | 1 (0 - 4) |  | 2 (0 - 4) | 2 (0 - 4) | 2 (0 - 4) | 2 (0 - 4) | 0.954 |

IQR: interquartile range; MDI: multiple daily injections of insulin; SMBG: self-monitoring of blood glucose; CGM: continuous glucose monitoring; SAP: sensor-augmented pump; PLGM: predictive low glucose management; HCL: hybrid closed loop; AHCL: advanced hybrid closed loop.

p refers to Kruskal-Wallis test. Data are expressed as median and interquartile range

**Table S3. Number of subjects with at least one episode of DKA or severe hypoglycaemia in the previous year by therapeutic strategies.**

|  | MDI+SMBG | MDI+CGM | SAP | PLGM | HCL/AHCL |
| --- | --- | --- | --- | --- | --- |
| DKA, n (%) | 0 | 1 (2.6) | 3 (3.1) | 2 (6.1) | 0 |
| Hypoglycaemia, n (%) | 13 (8.4) | 5 (12.8) | 6 (6.1) | 3 (9.1) | 6 (5.9) |

MDI: multiple daily injections of insulin; SMBG: self-monitoring of blood glucose; CGM: continuous glucose monitoring; SAP: sensor-augmented pump; PLGM: predictive low glucose management; HCL: hybrid closed loop; AHCL: advanced hybrid closed loop; DKA, diabetic ketoacidosis.

**Table S4. Confirmatory factor analysis**

| *CFI* | 0,86 |
| --- | --- |
| *TLI* | 0,82 |
| *RMSEA* | 0.10 (IC 95% 0.09-0.11) |

CFI: Comparative Fix Index; TLI: Tucker Lewis Index; RMSEA: Root Mean Square Error of Approximation

Structural integrity is considered good for values of CFI and TLI higher than 090, and for value of RMSEA lower than 0.05.

**Figure S1: Structure of the Italian DIDS version**


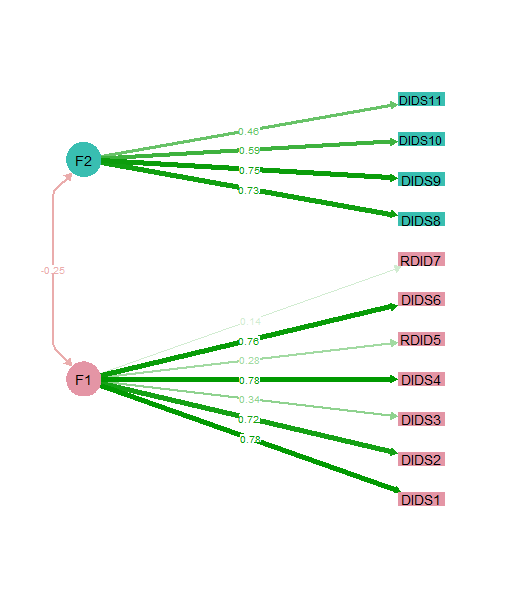


Figure S1, highlights that the Italian version of the questionnaire fits well with the two-domain structure of the original questionnaire.

DIDS , diabetes impact and device satisfaction.

**Table S5. Internal consistency**

| **DIDS domains** | | **median (1st Q; 3rd Q)** | **Cronbach's alpha α (95%CI)** |
| --- | --- | --- | --- |
| F1. Device Satisfaction | | 8 (8; 9) | 0.71 (0.66-0.75) |
|  | 1. Satisfaction | 8 (7; 9) | 0.63 (0.57-0.68) |
|  | 2. Trust | 9 (8; 9) | 0.65 (0.59-0.70) |
|  | 3. Helps me feel more in control of my diabetes | 9 (8; 10) | 0.70 (0.65-0.74) |
|  | 4. Helps me have good BG control | 8 (7; 9) | 0.64 (0.58-0.69) |
|  | 5. Easy to use | 8 (5; 10) | 0.74 (0.70-0.78) |
|  | 6. Is a hassle to use | 8 (8; 10) | 0.65 (0.59-0.70) |
|  | 7. Is too complicated | 10 (9; 10) | 0.71 (0.67-0.76) |
| F2. Diabetes Impact | | 2 (3; 5) | 0.74, CI: 0.70-0.78 |
|  | 8. Miss work/school/etc due to diabetes | 3 (1; 6) | 0.65 (0.58-0.70) |
|  | 9. Wake up at night to treat low BG | 3 (2; 6) | 0.63 (0.57-0.69) |
|  | 10. Worry about going low | 4 (2; 7) | 0.69 (0.64-0.74) |
|  | 11. Have a bad night's sleep due to diabetes | 1 (1; 3) | 0.74 (0.69-0.78) |

DIDS, diabetes impact and device satisfaction; 1st Q: first quartile; 3rd Q: third quartile

The two domains showed good internal consistency for both the domains; Cronbach's α ranged between 0.63 and 0.75 when assessed excluding single item.

**Figure S2. Discriminant analysis**

**
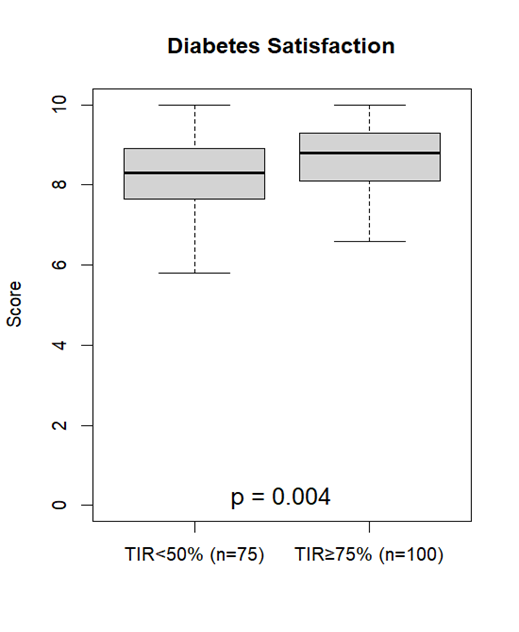

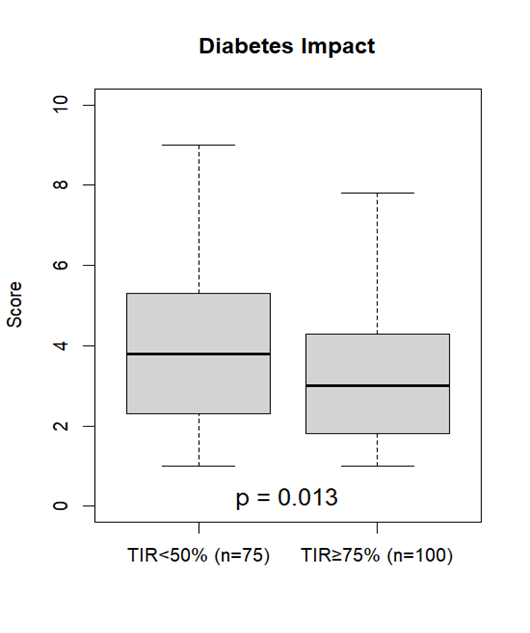
**

The test was performed comparing the DIDS scores between subjects with TIR values ≥ 75 % and subjects with TIR values < 50% showed significant differences in both DIDS domains. Patients with TIR values ≥ 75% had significantly higher satisfaction and lower impact than those with TIR values <50%.

DIDS, diabetes impact and device satisfaction; TIR, time in range.
